# Factors associated with under-five mortality in Bhutan: an analysis of the Bhutan National Health Survey 2012

**DOI:** 10.1186/s12889-018-6308-6

**Published:** 2018-12-17

**Authors:** Tashi Dendup, Yun Zhao, Deki Dema

**Affiliations:** 1 School of Public Health, Faculty of Health Sciences, Curtin University, Perth, WA 6102 Australia; 2 Population Wellbeing and Environment Research Lab (PowerLab), School of Health and Society, Faculty of Social Sciences, University of Wollongong, Wollongong, NSW 2522 Australia; 3 National Commission for Women and Children, Royal Government of Bhutan, Thimphu, Bhutan

**Keywords:** Under-five mortality, Bhutan, Total number of births, Sanitation, Household size

## Abstract

**Background:**

As an important marker for health equity and access, under-five mortality (UFM) is a primary measure for socioeconomic development. The importance of reducing UFM has been further emphasized in an ambitious target under Sustainable Development Goals. The factors influencing UFM are not adequately understood in Bhutan.

**Methods:**

The most recent dataset of the Bhutan National Health Survey (BNHS) 2012 was used in this study. Multiple logistic regression analysis using a backwards elimination approach was performed to identify significant factors influencing UFM. All statistical analyses were adjusted for the complex study design due to the multistage stratified cluster sampling used in BNHS.

**Results:**

Bhutan’s UFM rate was 37 per 1000 live births. The weighted mean age of the children was 7.3 years (SD: 1.53; range: 3–12). Mother’s age, household size, access to electricity and sanitation, residential region, and parity were the key factors associated with UFM. The UFM risk was significantly lower in children born to mothers aged 36–40 years, 41–45 years, and > 45 years when compared to that in children born to mothers aged < 26 years. The likelihood of mortality was 66% lower (95% CI: 0.21–0.55) among children born in households with > 5 members. Children born in households without electricity and safe sanitation had a significantly higher risk of death, by 81 and 49% respectively. Relative to those born in the west, children born in the central and eastern regions were 1.72 (95% CI: 1.07–2.77) and 2.09 (95% CI: 1.46–2.99) times more likely to die, respectively. Children born to mothers who gave birth to > 2 children were significantly more likely to die than their counterparts.

**Conclusion:**

These findings suggest that younger mother’s age, the higher number of births and being born in the central and eastern regions are associated with a higher UFM risk, whereas a larger household size and access to electricity and safe sanitation are key factors associated with lower UFM risk in Bhutan. Women empowerment, health education and strategies promoting maternal and child health in rural areas need to be scaled-up. Additionally, socioeconomic development programs should seek to reduce regional disparities.

**Electronic supplementary material:**

The online version of this article (10.1186/s12889-018-6308-6) contains supplementary material, which is available to authorized users.

## Background

Under-five mortality rate (UFMR) reflects the socioeconomic, health, and environmental conditions in which a child lives and develops [1]. It is defined as the probability of a child dying before attaining the exact age of 5 years, calculated per 1000 live births. Globally, UFMR has declined from 91 per 1000 live births in 1990 to 43 in 2015, which corresponds to a 52.7% reduction [1]. However, the world could not achieve the Millennium Development Goal (MDG) 4 of reducing UFMR by two-thirds in 2015 than in 1990 [1]. In 2015, more than 80% of the total 5.9 million under-five deaths was estimated to have occurred mostly from preventable causes in developing countries [1]. Under-five mortality rate as a socioeconomic and health barometer has thus been included in the Sustainable Development Goals (SDGs) with a renewed target of reducing the overall global mortality rate to less than 25 per 1000 live births by 2030 [2].

Bhutan is a small, landlocked, mountainous country situated in the Himalayas whose total projected population in 2016 was 768, 577 [3]. Healthcare services are provided free of charge through a healthcare system that is predominantly funded by the State. The private health service providers are limited to a few diagnostic centres and retail pharmacies [4]. Bhutan has been able to make significant progress in improving the health of its population. Bhutan’s UFMR declined from 134 per 1000 live births in 1990 to 37 in 2012 [5], and further to 33 per 1000 live births in 2015 [1], thus, achieving the MDG goal 4. Nonetheless, its current UFMR is still higher than that of some countries in the South Asian region such as Sri Lanka and the Maldives, which have a UFMR of around 9 to 10 per 1000 live births each, and with figures of 12 and 27 for Thailand and Indonesia, respectively [1]. Furthermore, out of more than 190 countries, Bhutan ranks 67th (in descending order) in terms of national UFMR [1].

Numerous studies have been conducted to examine the factors influencing under-five mortality (UFM) from countries in the South Asian region and around the world. Factors found to be associated with UFM include parent’s education, mother’s age, socioeconomic status, birth order, birth interval, and birth weight [6–18]. The place and region of residence, sex of the child, number of births in the past one year, and number of under-five children in a household are also documented to be associated with UFM [13–17, 19–21]. Furthermore, studies show that the place of delivery, previous death of siblings, marital status, household headship, and breastfeeding predicted child mortality risk [14–18, 20–23]. Evidence also suggests that UFM is correlated with household size, domestic violence, electricity, safe sanitation and drinking water, and indoor air pollution [6, 17, 21–29]. Use of health services and family planning services and methods are significantly associated with lower child mortality risk [7, 14, 15, 21, 23].

There is a paucity of literature on factors influencing UFM in Bhutan. The Global Burden of Disease Profile suggests malnutrition, being underweight, and household air pollution as major risk factors for child mortality [30]. The Bhutan Multiple Indicator Survey 2010 (BMIS) revealed a higher probability of death among male children, among children born in the eastern region, those born to mothers with low education level, as well as those born in poorer households and rural areas with an uneducated mother [31]. However, the BMIS neither comprehensively examined all probable factors nor carried out multiple regression analysis [31]. Likewise, the latest Bhutan National Health Survey (BNHS) conducted in 2012 did not assess the factors correlated with UFM [5]. The literature search did not reveal any study from Bhutan investigating the factors influencing UFM. To gain a better understanding of the factors influencing UFM that can help develop targeted and cost-effective strategies to improve child survival and reduce health disparities in Bhutan, this study aims to describe the distribution of UFM and to examine the key factors associated with UFM in Bhutan using the BNHS 2012 dataset. The use of stepwise hierarchical modelling and complex sample analysis that accounts for unequal selection probability enabled the identification of the significant factors.

## Methods

### Study design and sampling

This study used the latest nationally representative dataset (inclusive of all 20 districts) of the BNHS 2012 to explore the factors influencing UFM [5]. The BNHS 2012 was intended to generate nationally representative quality indicators and to assess the trend of population health. The main sampling strata were the urban and rural areas in each district, where the sample was selected in two stages. Villages in rural areas and blocks in the urban areas were selected systematically with probability proportional to size in each stratum. Households were listed and sequentially numbered in the selected primary sampling units. The households were then selected in each enumeration area using a systematic selection method.

The Ministry of Health (MoH) in collaboration with the National Statistics Bureau of Bhutan carried out the survey from November 2012 to February 2013. A total of 13,256 households (97%) were interviewed, where out of 45,635 eligible individuals aged 10–75 years, 39,789 (87%) were interviewed. For the domestic violence (females 10–75 years) and the women’s (10–49 years) questionnaires, the response rate was 90 and 91% respectively. Details of the methods and findings of the BNHS 2012 have been published previously [5].

### Merging data files and data extraction

In total, five BNHS 2012 datasets - birth history, women, household, immunization, and domestic violence data files were obtained from the MoH. The common base variables in all datasets were used to merge the women and the household datasets initially. The merged dataset was then merged with the birth history, immunization, and domestic violence datasets sequentially using the SPSS version 20 package. The final merged dataset was transferred to the STATA version 14 package for analysis [32].

To allow for the youngest child to have attained 5 years of life at the commencement of the BNHS in November 2012, and to be consistent with the BNHS 2012 report [5], all live births from November 2002 to October 2007 were extracted. Singleton births were extracted for our analysis to avoid potential confounding effect by multiple pregnancies. Out of 7136 total single births, 6397 were selected. Eight observations with no information on mortality were excluded, leaving a final sample of 6389 live births. This sample also included 221 and 310 births in 2002 and 2007 respectively, with missing information about the birth month.

The BNHS 2012 collected information on health-related variables only for births that occurred two years prior to the survey [5]. However, this study included births from November 2002 to October 2007. Thus, the shorter period in the provision of information on health-related variables resulted in a huge proportion (> 80%) of missing observations for these variables in the final merged dataset.

### Dependent variable

All deaths for boys and girls occurring before the exact age of 5 years from November 2002 to October 2007 were regarded as under-five death. To enable the regression analysis, the status of UFM of each child was further expressed as a binary variable; deaths and survivals were coded as 1 and 0 respectively.

### Conceptual framework for the study in Bhutan and independent variables

**Fig. 1:**
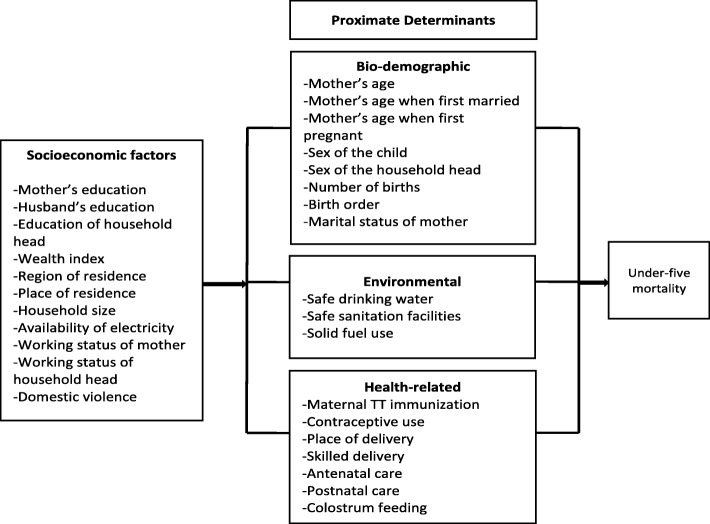
Conceptual framework illustrating the factors influencing under-five mortality

This study adapted and modified Mosley and Chen’s conceptual approach for analyzing determinants related to UFM in developing countries [33] in cognizance of the Bhutanese context, the significant factors found in the literature, and the availability of information in the BNHS 2012 dataset (Fig. 1).

The modified conceptual framework groups the independent variables into four categories: socioeconomic, bio-demographic, environmental, and health-related factors. Mother’s age, mother’s age when first married, mother’s age when first pregnant, mother’s marital status, sex of the child, the total number of births, sex of the household head, and birth order comprised the group of bio-demographic variables. The socioeconomic group included the education levels of the mother, husband, and the household head, the household size, the mother’s and household head’s working status, and the availability of electricity. Furthermore, this group included the household wealth index, region, place of residence, and the mother’s experience regarding domestic violence. Environmental variables considered were access to a safe drinking water source and sanitation facilities, and the use of solid fuel. Health-related factors comprised of the maternal tetanus toxoid (TT) immunization status, place of delivery, delivery by skilled attendants, antenatal and postnatal care, contraceptive use, and colostrum feeding. The operational definition of all these independent variables is provided in Table 1.

**Table 1:** Categories and operational definition of independent variables

	Variables	Definitions/categories
a	Bio-demographic variables	
1	Mother’s age	Age of the mother, in years (<=25, 26–30, 31–35, 36–40, 41–45, > 45)
2	Mother’s age when first married	Age of the mother when first married, in years (<=15, 16–20, 21–25, > 25)
3	Mother’s age when first pregnant	Age of the mother when she first conceived a child, in years (<=15, 16–20, 21–25, > 25)
4	Mother’s marital status	Marital status of the mother (married, not married (includes divorced, single, widowed, separated))
5	Sex of the child	Girl, Boy
6	Total births	Total number of births by the mother in her lifetime (<=2, 3–4, > 4)
7	Sex of the household head	Male, Female
8	Birth order	The birth order number/rank of the child in the family (first, second/third, > = fourth)
b	Socio-economic variables	
1	Mother’s education	Mother’s formal level of education/schooling (no education, primary, high school, tertiary (includes university, diploma, certificate), monastic/non-formal education)
2	Education of the husband	Husband’s formal level of education/schooling (no education, primary, high school, tertiary (includes university, diploma, certificate), monastic/non-formal education)
3	Education of the household head	Household head’s formal level of education/schooling (no education, primary, high school, tertiary (includes university, diploma, certificate), monastic/non-formal education)
4	Household size	Number of members in each household (<=5, > 5)
5	Electricity availability	Household has electricity (yes, no)
6	Mother’s working status	The working status of the mother (not working, working)
7	Household head’s working status	The working status of the household head (not working, working)
8	Wealth index	Economic/wealth status of the household (poorest, second, middle, fourth, richest)
9	Region of residence	The administrative region where the family resides (western, central, eastern)
10	Place of residence	Area of residence (urban, rural)
11	Domestic violence	Mother’s experience of domestic violence (no, yes)
c	Environmental variables	
1	Safe drinking water	Household has access to safe drinking water source (yes, no)
2	Safe sanitation facilities	Household has access to improved sanitation facilities (yes, no)
3	Use of solid fuel	Use solid fuel for cooking (yes, no)
d	Health-related variables	
1	Maternal TT immunization	Tetanus toxoid injection status of mothers who were or became pregnant in the past year preceding the survey (not immunized, partially immunized, fully immunized)
2	Contraceptive use	Use of contraceptives (ever used, never used)
3	Place of delivery	Place of delivery for the birth two years preceding the survey (institutions (includes health centres), home)
4	Skilled delivery	Delivery was attended by skilled persons for the child born two years preceding the survey (yes, no)
5	Antenatal care	Attended any number of antenatal care by the mother for the child born two years preceding the survey (yes, no)
6	Postnatal care	Attended any number of postnatal care by the mother for the child born two years preceding the survey (yes, no)
7	Fed colostrum	Fed colostrum soon after birth to the child born two years preceding the survey (yes, no)

### Statistical analysis

All statistical analyses were carried out using the STATA version 14 package [32]. STATA survey (svy) command for analyzing complex survey data was used in the data analysis of this study to take into account the complex survey design effect including clustering, stratification, and sampling weights of the BNHS 2012. The data analysis was conducted over three stages as is described below.

In Stage 1 of the univariate and bivariate analyses, the distribution of UFMR by the four groups of variables was obtained using cross-tabulations. Chi-square (χ2) tests and simple logistic regression analyses were performed. Crude (unadjusted) odds ratio (COR) and the respective 95% confidence interval (CI) were generated and reported.

Stage 2 involved employing multiple logistic regression (MLR) analysis to identify significant factors associated with UFM. A stepwise hierarchical modelling pertaining to the conceptual framework (Fig. 1) was performed for the four groups of variables, one by one and consecutively. To reduce the likelihood of missing important factors, varied significance levels were applied [34]. Initially, an MLR analysis was carried out for the group of socioeconomic variables, where any variables with a *p*-value of 0.20 or lower (*p* < 0.2) as found in the bivariate analyses were entered in the model. Based on this MLR analysis, the variables having a significant association with UFM at a 10% level (*p* < 0.1) were retained for subsequent modelling. The bio-demographic variables with a p-value of 0.20 or lower in the bivariate analyses were consequently added to the initial model and an MLR analysis was performed exploring the effect of the bio-demographic variables in the presence of significant socioeconomic variables. Variables that attained significance at a 10% level were further retained. The same approach was reiterated including environmental variables and subsequently retaining variables significant at a 10% level. Finally, the health-related variables with a *p*-value of 0.20 or lower, as found in the bivariate analyses, were entered and assessed for their effect in the presence of significant socioeconomic, bio-demographic, and environmental variables to build the final model.

Stage 3 (final stage) comprised of two additional regression modellings. The variables with a p-value of < 0.001 obtained in the bivariate analyses and the factors significantly associated with UFM as per the literature, were included in the final model obtained in Stage 2 to reassess their effect. Additionally, the possible influence of interaction between household size and other variables (wealth index, mother’s working status, mother’s education level, and place of residence) on UFM was tested.

At each level of the modellings, a backwards elimination regression approach was used to build a parsimonious model. Variables found to be significantly associated with UFM at a 5% significance level (with *p* < 0.05) were retained in the final model. The adjusted odds ratio (AOR) and the respective 95% CI were then reported. Variance inflation factor (VIF) was used to assess the multicollinearity among the independent variables at stage 2 and 3 modellings. An F-adjusted mean residual test (Stata survey command: svylogitgof) was used to assess the goodness of fit of the final model. Bootstrapping with 200 replications was used to validate the final model.

## Results

Out of 6389 singleton live births from November 2002 to October 2007, 258 died. This gave a weighted UFMR of 36.9 per 1000 live births. The estimates of UFMR and its distribution by the four groups of factors are provided in sections a), b), c) and d) under Table 2. The UFMR was found to be higher among male children, and among children whose mothers were older than 45 years, who were younger than 16 years when they first married, and had more than 4 births. The UFMR was found to be significantly higher among children born to parents without education and/or had monastic or non-formal education. Children born in households with less than 6 members, born in households without electricity, and born to non-working mothers experienced higher mortality rates. Furthermore, the UFMR was significantly higher among children born in poor households, in the eastern and the central regions, in households that used solid fuel, in households without safe sanitation facilities and among children living in rural areas. Significantly higher rate of UFM was additionally found among children whose mothers did not feed colostrum to their infants.

**Table 2:** Background characteristics and distribution of under-five mortality rate in Bhutan (*N* = 6389, *N*^a^ = 6209)

Variables	N	N^a^ (%)	UFMR^a^	*P*-value^b^
*a) Bio-demographic factors*				
Mother’s age (years)				0.442
<=25	573	597 (9.6)	40	
26–30	1893	1877 (30.2)	32	
31–35	1837	1748 (28.2)	43	
36–40	1245	1174 (18.9)	29	
41–45	595	594 (9.6)	40	
> 45	246	220 (3.5)	52	
Mother’s age when first married (years)				0.101
<= 15	837	857 (13.8)	47	
16–20	3867	3753 (60.5)	40	
21–25	1123	1076 (17.3)	24	
> 25	208	220 (3.6)	30	
Not reported/missing	354	302 (4.9)	28	
Mothers age when first pregnant (years)				0.551
<= 15	311	333 (5.4)	33	
16–20	3508	3434 (55.3)	39	
21–25	1906	1782 (28.7)	34	
> 25	381	360 (5.8)	28	
Not reported/missing	283	299 (4.8)	51	
Mother’s marital status				0.101
Married	5742	5678 (91.4)	38	
Not married	647	531 (8.6)	27	
Sex of the child				0.054
Girl	3192	3081 (49.6)	31	
Boy	3197	3128 (50.4)	43	
Total births				< 0.001
<=2 births	2072	2111 (34)	12	
3–4 births	2989	2838 (45.7)	42	
> 4 births	1327	1259 (20.1)	68	
Not reported/missing	1	0.7 (0.01)	0	
Sex of the household head				0.611
Female	2560	2213 (35.7)	36	
Male	3829	3995 (64.4)	38	
Birth order				0.101
First	1968	2006 (32.3)	29	
Second or third	2896	2732 (44)	36	
> = Fourth	1525	1471 (23.7)	49	
*b) Socioeconomic factors*				
Mother’s education				0.031
No education	3806	3662 (58.9)	43	
Primary	734	702 (11.3)	27	
High school	691	699 (11.3)	14	
Tertiary	89	121 (2)	17	
Monastic/NFE	1051	1014 (16.3)	41	
Not reported/missing	18	11 (0.2)	25	
Education of the husband				0.002
No education	2750	2704 (43.5)	45	
Primary	1192	1141 (18.4)	32	
High school	1015	1007 (16.2)	18	
Tertiary	230	262 (4.2)	11	
Monastic/NFE	842	789 (12.7)	56	
Not reported/missing	360	306 (4.9)	20	
Education of the household head				0.002
No education	3685	3467 (55.8)	41	
Primary	955	963 (15.5)	33	
High school	755	808 (13.0)	13	
Tertiary	206	223 (3.6)	9	
Monastic/NFE	771	740 (11.9)	59	
Not reported/missing	17	8 (0.1)	34	
Household size				0.098
<=5	3306	3348 (53.9)	43	
> 5	3083	2861 (46.1)	30	
Electricity availability				0.007
Yes	5602	5519 (88.9)	32	
No	782	681 (11)	77	
Not reported/missing	5	9 (0.1)	0	
Mother’s working status				0.013
Working	1906	1910 (30.8)	27	
Not working	4417	4295 (68.6)	42	
Not reported/missing	66	40 (0.6)	29	
Household head’s working status				0.685
Working	4311	4444 (71.6)	35	
Not working	2058	1748 (28.2)	41	
Not reported/missing	20	17 (0.3)	0	
Wealth index				< 0.001
Poorest	1407	1390 (22.4)	64	
Second	1286	1227 (19.8)	46	
Middle	1433	1289 (20.8)	34	
Fourth	1257	1230 (19.8)	22	
Richest	1006	1074 (17.3)	13	
Region of residence				< 0.001
Western	1913	2600 (41.9)	19	
Central	2385	1590 (25.6)	42	
Eastern	2091	2019 (32.5)	56	
Place of residence				< 0.001
Urban	1399	1489 (24)	16	
Rural	4990	4720 (76)	44	
Domestic violence				0.666
Yes	871	769 (12.4)	34	
No	5516	5438 (87.6)	37	
Not reported/missing	2	2 (0.2)	0	
*c) Environmental factors*				
Safe drinking water				0.092
Yes	6234	6043 (97.3)	37	
No	149	158 (2.5)	25	
Not reported/missing	6	9 (0.2)	22	
Safe sanitation facilities				< 0.001
Yes	4137	4067 (65.5)	27	
No	2242	2135 (34.4)	57	
Not reported/missing	10	7 (0.1)	0	
Use of solid fuel				< 0.001
Yes	1800	1826 (29.4)	55	
No	4588	4382 (70.6)	29	
Not reported/missing	1	0.2 (0.003)	0	
*d) Health-related factors*				
Maternal TT immunization				0.421
Not immunized	46	39 (0.6)	9	
Partially immunized	369	355 (5.7)	36	
Fully immunized	182	160 (2.6)	36	
Not reported/missing^c^	5792	5655 (91.1)	37	
Contraceptive use				0.055
Ever used	5365	5147 (82.9)	39	
Never used	1024	1062 (17.1)	29	
Place of delivery				0.087
Institutional	737	716 (11.5)	53	
Home	435	432 (7)	39	
Not reported/missing^c^	5217	5061 (81.5)	35	
Skilled delivery				0.101
Yes	749	726 (11.7)	52	
No	423	422 (6.8)	39	
Not reported/missing^c^	5217	5061 (81.5)	35	
Antenatal care				0.097
Yes	1141	1123 (18.1)	48	
No	31	25 (0.4)	0	
Not reported/missing^c^	5217	5061 (81.5)	35	
Postnatal care				0.060
Yes	805	811 (13.1)	42	
No	367	337 (5.4)	61	
Not reported/missing^c^	5217	5061 (81.5)	35	
Fed colostrum				0.006
Yes	1032	1017 (16.4)	45	
No	66	71 (1.1)	97	
Not reported/missing^c^	5291	5121 (82.5)	34	

^a^Weighted, UFMR = under-five mortality rate as ‘deaths per 1000 live births’, ^b^Chi-square (χ2) test, TT = tetanus toxoid, ^c^The shorter period in the provision of information on health-related variables by the Bhutan National Health Survey 2012 resulted in a huge proportion (> 80%) of missing observations for these variables in this study

Sections a), b), c) and d) in Table 3 highlight the crude and the adjusted odds ratios and their 95% CIs. Compared to children whose mothers were younger than 15 years when first married, those born to mothers aged 21–25 years when first married had significantly lower odds of dying. Children born to mothers who gave birth to more than 2 children had significantly higher odds of dying. Boys were 1.38 times more likely to die than girls (*p* = 0.055). The odds of UFM was significantly lower among children whose fathers had a high school and tertiary level of education and whose household head had received high school education. The likelihood of UFM was also significantly higher among children born in households without electricity, in the eastern and central regions, and those living in rural areas. The odds of UFM reduced significantly with increasing wealth quintiles and was lower among those born to working mothers. The likelihood of UFM was 2.18 and 1.95 times significantly higher among children born in households without safe sanitation and those that used solid fuel for cooking respectively. Colostrum feeding practice after birth was associated with UFM, whereas the rest of the health-related variables were not found to be significant.

**Table 3:** Factors associated with under-five mortality in Bhutan (crude/unadjusted and adjusted odds ratio)

Variables	COR	95% CI		*P*-value	AOR	95% CI		*P*-value
*a) Bio-demographic*								
Mother’s age (years)				0.189				< 0.001
<=25	1.00				1.00			
26–30	0.81	0.41	1.59	0.525	0.57	0.30	1.10	0.093
31–35	1.08	0.56	2.08	0.809	0.53	0.28	1.04	0.063
36–40	0.73	0.42	1.28	0.263	0.29	0.18	0.45	< 0.001
41–45	1.02	0.51	2.01	0.964	0.25	0.11	0.57	0.001
> 45	1.33	0.47	3.79	0.584	0.23	0.07	0.74	0.015
Mother’s age when first married (years)				0.031				
<=15	1.00							
16–20	0.84	0.59	1.19	0.317				
21–25	0.50	0.30	0.84	0.010				
> 25	0.64	0.20	1.99	0.434				
Mothers age when first pregnant (years)				0.630				
<=15	1.00							
16–20	1.17	0.52	2.61	0.706				
21–25	1.02	0.44	2.36	0.966				
> 25	0.84	0.31	2.25	0.725				
Mother’s marital status				0.103				
Married	1.00							
Not married	0.69	0.45	1.08	0.103				
Sex of the child				0.055				
Girl	1.00							
Boy	1.38	0.99	1.93	0.055				
Total births				< 0.001				< 0.001
<=2 births	1.00				1.00			
3–4 births	4.70	2.26	6.06	< 0.001	4.85	2.80	8.50	< 0.001
> 4 births	6.19	3.21	11.95	< 0.001	15.15	6.60	34.82	< 0.001
Sex of the household head				0.611				
Female	1.00							
Male	1.06	0.84	1.34	0.611				
Birth order				0.182				
First	1.00							
Second or third	1.27	0.91	1.78	0.165				
> = Fourth	1.71	0.96	3.05	0.068				
*b) Socioeconomic*								
Mother’s education				0.165				
No education	1.00							
Primary	0.62	0.34	1.16	0.133				
High school	0.32	0.13	0.79	0.014				
Tertiary	0.38	0.07	2.24	0.282				
Monastic/NFE	0.96	0.68	1.36	0.824				
Education of the husband				0.009				
No education	1.00							
Primary	0.69	0.45	1.06	0.089				
High school	0.38	0.20	0.73	0.004				
Tertiary	0.23	0.06	0.96	0.044				
Monastic/NFE	1.25	0.79	1.99	0.338				
Education of the household head				0.006				
No education	1.00							
Primary	0.82	0.52	1.29	0.382				
High school	0.31	0.13	0.77	0.013				
Tertiary	0.22	0.04	1.26	0.087				
Monastic/NFE	1.48	0.95	2.32	0.086				
Household size				0.100				< 0.001
<=5	1.00				1.00			
> 5	0.70	0.46	1.07	0.100	0.34	0.21	0.55	< 0.001
Electricity availability				< 0.001				0.026
Yes	1.00				1.00			
No	2.53	1.49	4.27	0.001	1.81	1.08	3.03	0.026
Mother’s working status				0.013				
Not working	1.00							
Working	0.64	0.45	0.90	0.012				
Household head’s working status				0.501				
Working	1.00							
Not working	1.17	0.73	1.88	0.501				
Wealth index				< 0.001				
Poorest	1.00							
Second	0.70	0.47	1.05	0.084				
Middle	0.51	0.32	0.81	0.005				
Fourth	0.33	0.22	0.49	< 0.001				
Richest	0.19	0.09	0.40	< 0.001				
Region of residence				< 0.001				< 0.001
Western	1.00				1.00			
Central	2.31	1.43	3.72	0.001	1.72	1.07	2.77	0.025
Eastern	3.11	2.04	4.73	< 0.001	2.09	1.46	2.99	< 0.001
Place of residence				< 0.001				
Urban	1.00							
Rural	2.89	1.74	4.81	< 0.001				
Domestic violence				0.667				
No	1.00							
Yes	0.90	0.55	1.47	0.667				
*c) Environmental*								
Safe drinking water				0.658				
Yes	1.00							
No	0.68	0.12	3.83	0.658				
Safe sanitation facilities				< 0.001				0.012
Yes	1.00				1.00			
No	2.18	1.62	2.95	< 0.001	1.49	1.09	2.03	0.012
Use of solid fuel				< 0.001				
No	1.00							
Yes	1.95	1.48	2.56	< 0.001				
*d) Health-related*								
Maternal TT immunization				0.603				
Not immunized	1.00							
Partially immunized	0.40	0.07	2.47	0.315				
Fully immunized	0.39	0.06	2.81	0.343				
Contraceptive use				0.056				
Ever used	1.00							
Never used	0.73	0.53	1.01	0.056				
Place of delivery				0.352				
Institutional	1.00							
Home	0.72	0.36	1.45	0.895				
Skilled delivery				0.410				
Yes	1.00							
No	0.75	0.37	1.52	0.410				
Antenatal care				NA				
Yes	NA	NA	NA	NA				
No	NA	NA	NA	NA				
Postnatal care				0.202				
Yes	1.00							
No	1.49	0.80	2.76	0.202				
Fed colostrum				0.042				
Yes	1.00							
No	2.26	1.03	4.97	0.042				

*COR* crude odds ratio*, AOR* adjusted odds ratio*, CI* confidence interval*, TT* tetanus toxoid*, NA* not available

The final adjusted model revealed the mother’s age, the total number of births, the region of residence, the household size, electricity, and safe sanitation as significant factors influencing UFM (Table 3). Compared to those born to younger (≤25 years) mothers, children born to mothers aged 36–40 years, 41–45 years, and more than 45 years had significantly lower odds of UFM. The odds of death among children born to mothers who gave birth to 3–4 or more than 4 children was significantly higher than those born to mothers with less than 3 children. Children born in households with at least 6 members had 66% lower odds of dying before reaching the age of 5 years than those born in households with less than 6 members (*p* < 0.001). The odds of UFM was significantly higher among children born in households without electricity (AOR = 1.81, *p* = 0.026) and those born in the central (AOR = 1.72, *p* = 0.025) and eastern (AOR = 2.09, *p* < 0.001) regions. The likelihood of UFM was 1.49 times higher for those children born in households without safe sanitation facilities than their counterparts (*p* = 0.012).

The possible influence of interaction between household size and other variables (wealth index, mother’s working status, mother’s education level, and place of residence) on UFM was found to be not significant, and are thus not reported. All VIFs were < 10, suggesting that multicollinearity was not a concern in the regression analysis. The F-adjusted mean residual goodness of fit test indicated no evidence for a lack of fit of the final model (*p* = 0.120). The bootstrap estimates were highly close to that of the final model supporting the internal validity of the final model (Additional file 1: Table S1).

## Discussion

To the best of our knowledge, this is the first study investigating the factors influencing UFM in Bhutan using the most recent nationally representative datasets of the BNHS 2012. The most significant predictors were, the total number of births, the household size, the region of residence, the mother’s age, safe sanitation, and electricity. The education level of the household head and the father, the mother’s age when first married and working status, the wealth index, the place of residence, solid fuel use, and colostrum feeding were also found to be significantly associated with UFM when not adjusted for other variables. Identification of these factors can be imperative in informing the design of evidence-based strategies aimed to improve child survival. The UFMR estimate in this study was 36.9 per 1000 live births (258 under-five deaths of the total 6389 singleton live births), while the BNHS 2012 reported 236 under-five deaths of the total 6237 live births giving a UFMR of 37 per 1000 live births [5]. The inclusion of births with the missing month of the birth year and restriction to singleton births in this study might have led to this marginal difference in the UFMR estimate.

This study found that the odds of UFM significantly decreased with the increase in mother’s age. The finding is consistent with other studies from India and Africa that found a lower likelihood of UFM among children of older mothers [15, 23, 35]. However, some studies showed a higher risk of child mortality in younger and older mothers [12, 25, 36]. A low level of education, pregnancy complications associated with waning reproductive system, higher parity and chronic illnesses typical of old age are possible explanations. Older mothers, nevertheless, may also be well prepared socially and mentally to care for their child. They could also be economically better, which could impact child health and development [37], possibly attributable to the higher use of maternal and child health services [38]. The finding indicates that children of young mothers are faced with higher mortality and suggests the need to intensify reproductive health education and family planning services.

This study also found that higher number of births were associated with a greater risk of UFM. Studies have also shown similar elevated risk of UFM among children born to mothers who gave more births [14, 15, 19]. Higher parity is often associated with shorter birth intervals, which influences UFM risk through depletion of mother’s health and nutritional status, and premature birth [39]. Pressure on resources in the household can also increase with an increase in the number of children potentially leading to undernutrition. Higher parity can be also related to the inadequate knowledge, availability, and use of family planning services. The contraceptive prevalence rate in Bhutan was 65.6% in 2010 [31] and the unmet needs of contraception for Bhutanese women aged 15–19 years was 27.4% [40]. Therefore, enhancing health education and access to health and family planning services may potentially improve child survival.

Consistent with findings of other studies [23, 27, 28, 36], this study also found that larger family size was associated with a lower risk of UFM. The findings support the view that larger households may have better resources such as more experienced child care providers and more working-age adults contributing to the household income. This relationship is plausible in the Bhutanese context where the tradition of extended family is still vibrant in the communities although this is changing gradually [41]. Additionally, a national survey found that one in every five people was a member of an extended family [42]. Besides, the household size as per the BNHS 2012 considers all persons living together and sharing living space and food arrangement including family resources. Thus, infants and small children usually live with a whole cluster of adults including older siblings who can take care of the child. In contrast, the higher risk of child death has been associated with larger family size [21]. Larger family size may indicate more children, leading to intra-sibling competition for limited resources and inadequate attention and care to children heightening their mortality risk. However, the finding in this study suggests that larger family size is protective against UFM in Bhutan, possibly through more people to care for the child and resources.

In this study, children born in the eastern and central region had significantly higher UFM risk than those born in the western region. The result supports the findings of BMIS 2010 that also revealed a higher risk of child deaths in the eastern region [31]. This can be explained partly by the high incidence of poverty particularly in rural areas of the eastern and central districts of Bhutan, and of malnutrition, poor sanitation and low health knowledge in eastern Bhutan [31, 42–44]. Equally, the districts in the western region were found to have a far greater chance of escaping hunger, possibly from the accessibility to health and public services [44]. These indicate that equitable regional socioeconomic development may help reduce regional disparities in child survival and reduce UFM mortality.

The availability of electricity in the households emerged to be a significant protective factor against UFM in Bhutan. This finding is consistent with results from other studies [17, 25, 26]. The availability of electricity could enable the access of radio and television to facilitate health awareness and promote hygienic practices (such as refrigeration of foods) that can reduce infectious diseases (such as diarrhoea) among children. Availability of electricity may also indicate a higher social status of a household [17]. Besides, it may potentially enhance income from longer working hours after dusk, particularly in the rural areas, and improve indoor air quality through decreased use of solid fuels thereby contributing towards reduction of respiratory infections including pneumonia in children. As expected, the probability of UFM was significantly lower in the households with safe sanitation facilities. The coverage of safe sanitation in Bhutan was low at 58.4% [31]. Furthermore, the effect of inadequate sanitation is reflected in the high prevalence of diarrhea, malnutrition, and respiratory infections [5, 31, 45]. Both the results imply the need for interventions to further promote and improve sanitation and hygiene and increase electricity coverage.

### Policy implications

The findings of this study suggest that improving sanitation and hygiene, access to electricity, family planning services, reproductive and health services, and ensuring regional equitable development can improve child survival. The findings can be useful in devising focused strategies to improve child health and reduce health inequalities in Bhutan. This is expected to help the child and related health programs to identify the at-risk population essential to target interventions and channel resources. The findings can also assist advocacy for equitable regional development and in designing educational programs. Likewise, the results can support resource mobilization efforts aimed to improve sanitation, family planning and health education. The present study also serves as a basis for future studies and in evaluating child health policies.

### Strengths and limitations of the study

This study is a first of its kind to be undertaken in Bhutan using a nationally representative dataset. The large sample size, a high response rate, and the use of valid survey methods lends to the study’s credibility. Moreover, the availability of a wide range of variables permitted deeper investigation of numerous factors. Additionally, the use of complex survey data analysis with a stage-wise regression modelling approach allays the fear of drawing biased inferences arising from multi-stage stratified sampling.

This study also had the following limitations. Firstly, the cross-sectional design of the study prevents drawing causal inferences on the associations revealed. The demographic and health surveys are retrospective studies. Thus, they are prone to recall bias in remembering birth history and mortality events. These surveys only interviewed mothers who are alive, which could have led to the underestimation of UFMR and the effect of various factors. Furthermore, due to the non-availability of information, the effect of a few factors such as birth weight and/or size, birth interval, and polygamy could not be assessed. The lack of information for a large number of observations for health-related variables could have restricted this study in examining their effect on UFM. The beneficial effects of health factors are incontrovertible and thus need to be investigated in future studies. Finally, the backwards elimination regression approach used in this study may underestimate the importance of certain combinations of variables and also has the potential to identify spurious associations arising from model overfitting [46].

## Conclusions

Based on the dataset of BNHS 2012, the weighted UFMR in Bhutan was 36.9 per 1000 live births in 2012. Mother’s age, region of residence, total number of births, household size, access to electricity, and safe sanitation were significantly associated with UFM. Under-five mortality is still an important public health concern in Bhutan and the factors influencing it are varied. Intensified efforts are required to address these factors with targeted interventions to achieve the SDGs by 2030. Empowerment of women through education and wider health education coverage, particularly in rural and lesser inaccessible areas, will have a broader impact on child survival by improving the socioeconomic status, health awareness and health service utilization. Programs should promote breastfeeding and family planning services, as well as motivate the use of health service and positive health behaviours. Additional efforts to promote sanitation and hygiene and to increase access to mother and child health services in rural and lesser accessible areas should be prioritized. Strategies aimed towards ensuring equitable regional socioeconomic development may further impact child survival.

Additional studies including information on birth interval, birth size/weight, and health-related factors are required. Factors impacting infant mortality also need to be examined, given that mortality is likely to be concentrated in this group with improvements in child survival. Similar future studies can thus enable the comparison of factors and in assessing improvements in health inequalities over time. Doing so can help inform strategies most relevant to the changing epidemiology of child survival in Bhutan.

## Additional file

Additional file 1: **Table S1.** Comparison of odds ratio estimates in final model with bootstrapped estimates from 200 replications. (DOCX 19 kb)

## Abbreviations

AOR: Adjusted odds ratio
BMIS: Bhutan Multiple Indicator Survey
BNHS: Bhutan National Health Survey
CI: Confidence interval
COR: Crude odds ratio
MDG: Millennium Development Goal
MLR: Multiple Logistic Regression
MoH: Ministry of Health
SDG: Sustainable Development Goal
TT: Tetanus Toxoid
UFM: Under-five mortality
UFMR: Under-five mortality rate

## References

[1] United Nations Children's Fund (2016). The State of The World's Children 2016: A fair chance for every child.

[2] United Nations (2015). Sustainable development goals: 17 goals to transform our world.

[3] National Statistics Bureau. Statistical Year Book of Bhutan 2016. Thimphu, Bhutan: Royal Government of Bhutan. http://www.nsb.gov.bt/publication/publications.php?id=3. Accessed 21 Aug 2018.

[4] Sharma J, Zangpo K, Grundy J (2014). Measuring universal health coverage: a three-dimensional composite approach from Bhutan. WHO South East Asia J Public Health.

[5] Ministry of Health. National Health Survey Report 2012. Thimphu, Bhutan: Royal Government of Bhutan. http://www.health.gov.bt/publications/national-health-survey/. Accessed 21 Aug 2018.

[6] Akinyemi JO, Bamgboye EA, Ayeni O (2013). New trends in under-five mortality determinants and their effects on child survival in Nigeria: a review of childhood mortality data from 1990-2008. Afr Popul Stud.

[7] Chowdhury AH (2013). Determinants of under-five mortality in Bangladesh. Open Journal of Statistics.

[8] Dadi AF (2015). A systematic review and meta-analysis of the effect of short birth interval on infant mortality in Ethiopia. PLoS One.

[9] Ezeh OK, Agho KE, Dibley MJ, Hall JJ, Page AN (2015). Risk factors for postneonatal, infant, child and under-5 mortality in Nigeria: a pooled cross-sectional analysis. BMJ Open.

[10] Hossain MM, Mani KK, Islam MR (2015). Prevalence and determinants of the gender differentials risk factors of child deaths in Bangladesh: evidence from the Bangladesh demographic and health survey. 2011 PLoS Negl Trop Dis.

[11] Huda TM, Tahsina T, El Arifeen S, Dibley MJ. The importance of intersectoral factors in promoting equity-oriented universal health coverage: a multilevel analysis of social determinants affecting neonatal infant and under-five mortality in Bangladesh. Glob Health Action. 2016;9. 10.3402/gha.v9.29741. 10.3402/gha.v9.29741 PMC4754013 26880153

[12] Kanmiki EW, Bawah AA, Agorinya I, Achana FS, Awoonor-Williams JK, Oduro AR (2014). Socio-economic and demographic determinants of under-five mortality in rural northern Ghana. BMC Int Health Hum Rights.

[13] Dejene T, Girma E (2013). Social determinants of under-five mortality in Ethiopia: Event history analysis using evidence from Ethiopian Demographic and Health Survey (EDHS). Health.

[14] Adhikari R, Podhisita C (2010). Household headship and child death: evidence from Nepal. BMC Int Health Hum Rights.

[15] Abir T, Agho KE, Page AN, Milton AH, Dibley MJ (2015). Risk factors for under-5 mortality: evidence from Bangladesh demographic and health survey, 2004–2011. BMJ Open.

[16] Mani K, Dwivedi SN, Pandey RM (2012). Determinants of under-five mortality in rural empowered action group states in India: an application of cox frailty model. Int J MCH AIDS.

[17] Karmaker S, Lahiry S, Roy D, Singha B (2014). Determinants of infant and child mortality in Bangladesh: time trends and comparisons across South Asia. Bangladesh Journal of Medical Science.

[18] Singh R, Tripathi V (2013). Maternal factors contributing to under-five mortality at birth order 1 to 5 in India: a comprehensive multivariate study. Springerplus.

[19] Lee H-Y, Van Do D, Choi S, Trinh OTH, To KG (2016). Trends and determinants of infant and under-five childhood mortality in Vietnam, 1986–2011. Glob Health Action.

[20] Nasejje JB, Mwambi HG, Achia TN (2015). Understanding the determinants of under-five child mortality in Uganda including the estimation of unobserved household and community effects using both frequentist and Bayesian survival analysis approaches. BMC Public Health.

[21] Kayode GA, Adekanmbi VT, Uthman OA (2012). Risk factors and a predictive model for under-five mortality in Nigeria: evidence from Nigeria demographic and health survey. BMC Pregnancy Childbirth.

[22] Worku Z. Factors that affect under-five mortality among south African children: analysis of the south African demographic and health survey data set. In proceedings of the world congress on engineering and computer science: 20-22. San Francisco; 2009.

[23] Singh R, Tripathi V. Under-five mortality among mothers employed in agriculture: findings from a nationally representative sample. PeerJ. 2015;3. 10.7717/peerj.710. 10.7717/peerj.710 PMC4304864 25653900

[24] Ezeh OK, Agho KE, Dibley MJ, Hall JJ, Page AN (2014). The effect of solid fuel use on childhood mortality in Nigeria: evidence from the 2013 cross-sectional household survey. Environ Health.

[25] Charmarbagwala R, Ranger M, Waddington H, White H (2004). The determinants of child health and nutrition: a meta-analysis.

[26] Wang L (2003). Determinants of child mortality in LDCs: empirical findings from demographic and health surveys. Health Policy.

[27] Uddin J, Hossain Z (2008). Predictors of infant mortality in a developing country. Asian Journal of Epidemiology.

[28] Pandey MK. Maternal health and child mortality in rural India. http://mpra.ub.uni-muenchen.de/15927/ (2009). Accessed 01 Aug 2017.

[29] Hossain MA, Sumi NS, Haque ME, Bari W (2013). Consequences of intimate partner violence against women on under-five child mortality in Bangladesh. J Interpers Violence.

[30] Institute for Health Metrics and Evaluation. Global Burden of Disease (GDB) Profile: Bhutan. 2010. https://www.healthdata.org/sites/default/files/files/country_profiles/GBD/ihme_gbd_country_report_bhutan.pdf. Accessed 01 Aug 2017.

[31] National Statistics Bureau. Bhutan Multiple Indicator Survey 2010. Thimphu, Bhutan: Royal Government of Bhutan. http://www.nsb.gov.bt/publication/publications.php?id=1. Accessed 01 Aug 2017.

[32] StataCorp (2015). Stata Statistical Software: Release 14. College Station, TX, StataCorp LP.

[33] Mosley WH, Chen LC. An analytical framework for the study of child survival in developing countries. Popul Dev Rev 1984;10. Suppl:25–45. PMC2572391 12756980

[34] Bursac Z, Gauss CH, Williams DK, Hosmer DW (2008). Purposeful selection of variables in logistic regression. Source Code Biol Med.

[35] Ettarh RR, Kimani J (2012). Determinants of under-five mortality in rural and urban Kenya. Rural Remote Health.

[36] Kaldewei C (2010). Determinants of infant and under-five mortality–the case of Jordan.

[37] Sutcliffe AG, Barnes J, Belsky J, Gardiner J, Melhuish E. The health and development of children born to older mothers in the United Kingdom: observational study using longitudinal cohort data. BMJ. 2012;345. 10.1136/bmj.e5116 PMC3424227 22915663

[38] Reynolds HW, Wong EL, Tucker H (2006). Adolescents' use of maternal and child health services in developing countries. Int Fam Plan Perspect.

[39] Hobcraft JN, McDonald JW, Rutstein SO (1985). Demographic determinants of infant and early child mortality: a comparative analysis. Popul Stud.

[40] World Health Organization. Bhutan and Family Planning: An Overview. 2015. http://www.searo.who.int/entity/maternal_reproductive_health/documents/family-planning/en/. Accessed 01 Aug 2017.

[41] Barth F, Wikan U. Situation of children in Bhutan: an anthropological perspective: Centre for Bhutan Studies; 2011.

[42] National Statistics Bureau. Bhutan Living Standards Survey 2012 Report. Thimphu, Bhutan: Royal Government of Bhutan. http://www.nsb.gov.bt/publication/files/pub1tm2120wp.pdf. Accessed 21 Aug 2018.

[43] Mehta S (2007). Inter-regional variations in the inequality and poverty in Bhutan. Journal of Bhutan Studies.

[44] National Statistics Bureau. Bhutan Poverty Assessment 2014. Thimphu, Bhutan: Royal Government of Bhutan. http://www.worldbank.org/content/dam/Worldbank/document/SAR/bhutan-poverty-assessment.pdf. Accessed 21 Aug 2018.

[45] Ministry of Health. Annual health bulletin 2015. Thimphu, Bhutan: Royal Government of Bhutan http://www.health.gov.bt/publications/annual-health-bulletins/. Accessed 21 Aug 2018.

[46] Livingston E, Cao J, Dimick JB (2010). Tread carefully with stepwise regression. Arch Surg.

