# Supplementary material for: Factors associated with under-five mortality in Bhutan: an analysis of the Bhutan National Health Survey 2012
Source: BMC Public Health. 2018 Dec 17;18:1375. doi: 10.1186/s12889-018-6308-6 (PMC6296032; doi:10.1186/s12889-018-6308-6)
Supplement: Supplementary file 1 — Table S1. Comparison of odds ratio estimates in final model with bootstrapped estimates from 200 replications. (DOCX 19 kb) [file 12889_2018_6308_MOESM1_ESM.docx]

**Additional file 1: Table S1** Comparison of odds ratio estimates in final model with bootstrapped estimates from 200 replications

| **Variables** | **AOR*** | **95% CI** | | **P-value** | **AOR^#^** | **95% CI** | | **P-value** |
| --- | --- | --- | --- | --- | --- | --- | --- | --- |
| ***a) Bio-demographic*** | | | | | | | | |
| **Mother’s age (years)** |  |  |  |  |  |  |  |  |
| <=25 | 1.00 |  |  |  | 1.00 |  |  |  |
| 26-30 | 0.57 | 0.30 | 1.10 | 0.093 | 0.57 | 0.27 | 1.19 | 0.135 |
| 31-35 | 0.53 | 0.28 | 1.04 | 0.063 | 0.53 | 0.25 | 1.12 | 0.097 |
| 36-40 | 0.29 | 0.18 | 0.45 | <0.001 | 0.29 | 0.16 | 0.51 | <0.001 |
| 41-45 | 0.25 | 0.11 | 0.57 | 0.001 | 0.25 | 0.11 | 0.59 | 0.001 |
| >45 | 0.23 | 0.07 | 0.74 | 0.015 | 0.23 | 0.07 | 0.79 | 0.020 |
| **Total births** |  |  |  |  |  |  |  |  |
| <=2 births | 1.00 |  |  |  | 1.00 |  |  |  |
| 3-4 births | 4.85 | 2.80 | 8.50 | <0.001 | 4.85 | 2.89 | 8.14 | <0.001 |
| >4 births | 15.15 | 6.60 | 34.82 | <0.001 | 15.15 | 6.73 | 34.10 | <0.001 |
| ***b) Socioeconomic*** | | | | | | | | |
| **Household size** |  |  |  |  |  |  |  |  |
| <=5 | 1.00 |  |  |  | 1.00 |  |  |  |
| >5 | 0.34 | 0.21 | 0.55 | <0.001 | 0.34 | 0.20 | 0.56 | <0.001 |
| **Electricity availability** |  |  |  |  |  |  |  |  |
| Yes | 1.00 |  |  |  | 1.00 |  |  |  |
| No | 1.81 | 1.08 | 3.03 | 0.026 | 1.81 | 1.02 | 3.20 | 0.043 |
| **Region of residence** |  |  |  |  |  |  |  |  |
| Western | 1.00 |  |  |  | 1.00 |  |  |  |
| Central | 1.72 | 1.07 | 2.77 | 0.025 | 1.72 | 1.06 | 2.79 | 0.027 |
| Eastern | 2.09 | 1.46 | 2.99 | <0.001 | 2.09 | 1.43 | 3.04 | <0.001 |
| ***c) Environmental*** | | | | | | | | |
| **Safe sanitation facilities** |  |  |  |  |  |  |  |  |
| Yes | 1.00 |  |  |  | 1.00 |  |  |  |
| No | 1.49 | 1.09 | 2.03 | 0.012 | 1.49 | 1.08 | 2.05 | 0.014 |

*AOR*=adjusted odds ratio in the final model, AOR^#^=bootstrapped adjusted odds ratio, CI=confidence interval*
